# Genome-Wide Identification and Characterization of *TaCRY* Gene Family and Its Expression in Seed Aging Process of Wheat

**DOI:** 10.3390/cimb47070522

**Published:** 2025-07-06

**Authors:** Guoqing Cui, Xiuyan Cui, Junjie Wang, Menglin Lei, Xia Liu, Yanzhen Wang, Haigang Wang, Longlong Liu, Zhixin Mu, Xia Xin

**Affiliations:** 1 Center for Agricultural Genetic Resources Research, Key Laboratory of Crop Gene Resources and Germplasm Enhancement on Loess Plateau, Shanxi Agricultural University, Ministry of Agriculture and Rural Affairs, Taiyuan 030031, China; 2 Key Laboratory of Evaluation and Utilization of Crop Gene Resources, Institute of Crop Sciences, Chinese Academy of Agricultural Sciences, Ministry of Agriculture and Rural Affairs of China, Beijing 100081, China

**Keywords:** wheat, genome-wide, *TaCRY*, gene family, seed vigor

## Abstract

Cryptochromes (CRYs), as essential blue-light photoreceptors, play pivotal roles in modulating plant growth, development, and stress responses. Although *CRY*-mediated light signaling has been extensively studied in model species, their functions remain limited in wheat. In this work, a comprehensive analysis of the *TaCRY* gene family was performed in wheat, identifying 12 *TaCRY* members localized to distinct chromosomes 2, 6, and 7. TaCRYs contain the conserved PHR and CCT domains and diverse gene structures. Collinearity relationships indicated their dynamic evolution patterns during polyploidization. *Cis*-acting elements of *TaCRY* members associated with light responsiveness, phytohormone signaling, and abiotic stress were also identified. Transcriptome analysis revealed that the differential expression patterns of *TaCRY* members under seed vigor process. This study expands our understanding of *TaCRY* diversity and provides valuable molecular information for marker-assisted selection in wheat improvement.

## 1. Introduction

Cryptochromes (CRYs), a class of blue-light photoreceptors, are widely distributed in plants, animals, and microorganisms. In plants, *CRYs* regulate multiple aspects of the plant life cycle by modulating blue-light-dependent gene expression, including the inhibition of hypocotyl elongation, the promotion of cotyledon expansion, flowering regulation, the suppression of the germination of dormant seeds, and so on [1,2].

*CRYs* were first discovered in the model plant *Arabidopsis thaliana* [3], and plant *CRYs* comprise three distinct isoforms: *CRY1*, *CRY2*, and *CRY3*. *CRY1*, also known as the *long hypocotyl 4* (*HY4*), encodes a DNA photolyase-related protein essential for the blue-light inhibition of the hypocotyl elongation of *Arabidopsis* seedlings, the promotion of cotyledon opening, and seedling de-etiolation [4]. *CRY2*, initially identified for its role in flowering time regulation, exhibits partial functional redundancy with *CRY1* but also governs distinct biological functions in blue-light responses [5,6]. *CRY3*, an atypical CRY-DASH-type subfamily member localized to mitochondria and chloroplasts, displays no observable phenotypic changes under various visible light conditions in *Arabidopsis thaliana*, rendering its physiological significance enigmatic [7,8]. Structurally, plant CRYs contain two typical core domains: the highly conserved N-terminal photolyase homology region (PHR domain) and the variable C-terminal extension (CCE domain). The PHR domain (~500 amino acids in length) harbors a flavin adenine dinucleotide (FAD) chromophore-binding site to mediate blue-light signal perception and is crucial for CRY dimerization. The CCE domain facilitates blue-light signal transduction through interactions with downstream effector proteins [9,10,11,12]. Mechanistically, CRYs adopt distinct conformational states depending on illumination. Under dark conditions, monomeric CRYs maintain an autoinhibited closed conformation via intramolecular interactions between the unphosphorylated CCE and PHR domains. A recent study revealed that monomeric CRY2 forms a CRY2-FLs inhibitory complex with FLAVIN-BINDING KELCH REPEAT F-BOX (FL) proteins (FL1/FL3), inhibiting FLs activity and consequently suppressing cell division and impeded root growth [13]. Upon exposure to blue light, the CCE domain undergoes light-dependent phosphorylation, causing electrostatic repulsion from the negatively charged PHR domain surface. Concurrently, the PHR domain undergoes homodimerization. This dual change converts CRYs from inactive to their photoactivated state [14,15]. The photoactivated CRYs propagate signals through interactions with diverse downstream effectors, including CIB transcription factors, BIC negative regulators, CIS1 kinases, COP1 ubiquitin ligases, PIF4 basic helix–loop–helix factors, PPK kinases, and so on [16,17,18,19,20,21,22], and these interactions propagate the blue-light signal through multiple pathways, ultimately integrating photoperception with developmental programs and stress responses to optimize plant fitness under fluctuating light environments.

The timely dormancy and germination of seeds are pivotal for determining plant population regeneration and crop yield, governed by a complex interplay of endogenous developmental programs and exogenous environmental cues [23]. Among these, light serves as a pivotal environmental signal that exerts significant influence on seed dormancy and germination [24,25]. Early studies demonstrated that red light promotes seed germination, whereas far-red light inhibits this process. Further research revealed that red light activates phytochrome B (PHYB), converting it to its bioactive Pfr form. The activated phyB-Pfr form interacts with downstream effectors, such as PIF1 transcription factors and REVEILLE1/2 (RVE1/2), triggering PIF1 ubiquitination and degradation via the 26S proteasome system, thereby relieving repression of germination-promoting genes and facilitating seed germination [26]. In darkness, phyB reverts to the inactive phyB-Pr form, allowing PIF accumulation and restoring seed dormancy. This phytochrome-mediated seed dormancy and germination is closely associated with the levels and signaling intensity of gibberellin (GA) and abscisic acid (ABA) balance. For instance, red light suppresses ABA biosynthesis via phyB and reduces ABA content to alleviate dormancy, while simultaneously promoting GA synthesis and activating GA signaling pathways to stimulate germination. This light–hormone synergistic regulation ensures germination under favorable environmental conditions [27,28,29]. Beyond phytochromes, *CRYs* also contribute to seed vigor regulation through distinct mechanisms. In barley, *CRY1* enhances the transcription of ABA biosynthesis genes and suppresses the expression of ABA catabolic genes, thereby increasing ABA accumulation and inhibiting barley seed germination [30,31,32]. This *CRY*-mediated pathway provides an additional layer of light quality discrimination, ensuring germination occurs under optimal spectral conditions.

Wheat seeds inevitably lose vigor and quality during storage, leading to seed quality deterioration and reduced germination potential. Although *CRY*-mediated regulation of seed dormancy has been documented, wheat *TaCRY* members’ modulation of seed vigor lacks systematic investigation. This study conducted a genome-wide identification of the *TaCRY* gene family and analyzed the phylogenetic relationships, gene structures, conserved domains, collinearity, *cis*-regulatory elements, and transcriptional responses to wheat seed vigor during seed aging. The findings establish an important theoretical foundation for elucidating the molecular mechanisms underlying wheat seed vigor regulation by *TaCRYs* and provide valuable molecular information for future wheat breeding.

## 2. Materials and Methods

### 2.1. Identification of TaCRY Members

Whole-genome sequencing data of the cultivars Chinese Spring (*Triticum aestivum* L.), rice (*Oryza sativa* Japonica), maize (*Zea mays* L.), and foxtail millet (*Setaria italica* L.), encompassing reference genome sequences, protein sequences, and GFF3 annotation files, were sourced from the Ensembl Plants database (https://plants.ensembl.org/index.html, accessed on 20 May 2024). Protein sequences corresponding to three *AtCRY* members in *Arabidopsis thaliana* (Supplementary Table S1) were retrieved from the National Center for Biotechnology Information (NCBI) database (https://www.ncbi.nlm.nih.gov/, accessed on 8 January 2025). The sequences of *AtCRY* members served as query templates in a BLASTP search conducted via TBtools-II (version 2.313) software [33] to identify potential *TaCRY*, *OsCRY*, *ZmCRY,* and *SiCRY* members. Hidden Markov Models (HMMs) for the conserved domains PF00875, PF03441, and PF12546 of *CRY* members were downloaded from the Pfam database (http://pfam.xfam.org/, accessed on 15 January 2025) and utilized with the “simple HMM search” tool in TBtools-II (version 2.313) software [34] to screen and compare whole-genome protein sequences against these HMM profiles. Validation of *CRY* candidates was further performed using the NCBI-CDD tool (https://www.ncbi.nlm.nih.gov/Structure/bwrpsb/bwrpsb.cgi, accessed on 18 January 2025) [35,36,37]. By integrating results from BLASTP, HMMER, and NCBI-CDD analyses, *TaCRY* members were definitively confirmed using the Triticeae-GeneTribe platform (http://wheat.cau.edu.cn/TGT/, accessed on 25 January 2025) with functional annotation support [38].

### 2.2. Physicochemical Properties Analysis of TaCRY Members

The protein sequences of the *TaCRY* members were submitted to the “Protein Parameter Calculator” module in TBtools-II (version 2.313) software to predict key physicochemical attributes, including the number of amino acids (AA), molecular weight (MW), theoretical isoelectric point (pI), instability index, aliphatic index, and grand average of hydropathicity (GRAVY). The subcellular localization of TaCRY members was predicted using three tools: WOLF PSORT (http://wolfpsort.hgc.jp/, accessed on 26 January 2025), BUSCA (http://busca.biocomp.unibo.it/, accessed on 26 January 2025) [39], and Plant-mPLoc (http://www.csbio.sjtu.edu.cn/bioinf/plant-multi/, accessed on 26 January 2025) [40].

### 2.3. Phylogenetic Analysis of TaCRY Members

To elucidate the evolutionary relationships among the identified members, a total of twelve TaCRY, three AtCRY, four OsCRY, five ZmCRY, and four SiCRY, protein sequences were aligned using ClustalW within MEGA 7.0.26 software [41]. The neighbor-joining (NJ) method was used to construct a phylogenetic tree with the bootstrap value set to 1000, using the Poisson correction model and pairwise deletion. The online iTOL (http://itol.embl.de/, accessed on 30 January 2025) tool [42] was employed to further modify and visualize the phylogenetic tree.

### 2.4. Gene Motif, Conserved Domain and Structure Analysis of TaCRY Members

To systematically characterize the structural features of *TaCRY* members, we performed a multi-layered analysis integrating motif discovery, domain prediction, and gene structure visualization. The motifs of TaCRY members were identified using the MEME Suite v5.5.7 (https://meme-suite.org/meme/, accessed on 31 January 2025) [43], with the maximum number of motifs set to 10 and default parameters for all other settings. Conserved domains were predicted using the Batch CD Search tool available on the NCBI platform. Gene structure visualizations (exons, introns, and UTRs) was generated using the “Gene Structure View (Advanced)” module in TBtools-II (version 2.313) software, which integrates GFF3 annotations with genomic sequences. All structural features were consolidated and visualized using TBtools-II (version 2.313) software to enable comparative analysis. The tertiary structures of CRY proteins from *Arabidopsis thaliana* and wheat were predicted by AlphaFold3 (version 3.0.0), and the resulting models were subsequently visualized and analyzed with PyMOL (version 2.5.5) software.

### 2.5. Chromosomal Mapping and Collinearity Analysis of TaCRY Members

Chromosomal mapping of *TaCRY* members was performed using TBtools-II (version 2.313) software, based on positional information extracted from GFF3 files. Gene duplication events within the *TaCRY* gene family were analyzed using MCScanX module in TBtools-II (version 2.313) software and visualized using “Advanced Circos” tool in TBtools-II (version 2.313) software.

### 2.6. Cis-Acting Elements Analysis of TaCRY Members

To investigate the transcriptional regulation of *TaCRY* members, we analyzed promoter regions spanning 2000 bp upstream of the translation start codon (ATG) using the reference wheat genome. The promoter regions were then scanned for *cis*-acting elements using the PlantCARE database (https://bioinformatics.psb.ugent.be/webtools/plantcare/html/, accessed on 10 February 2025) [44]. Elements associated with plant growth and development, phytohormone responsiveness, and biotic/abiotic stress responses were identified and characterized. The spatial distribution of these regulatory elements across *TaCRY* promoters was visualized using TBtools-II (version 2.313) software to enable comparative analysis of transcriptional control features.

### 2.7. Expression Analysis of TaCRY Members

The RNA-seq expression profiles of *TaCRY* members were mined from the transcriptome data by Liang et al. [45]. The transcripts per million (TPM) value was used to normalize RNA-seq data. A Venn diagram was generated using the InteractiVenn website (https://www.interactivenn.net/, accessed on 22 February 2025) [46].

### 2.8. RNA Extraction and RT-qPCR Analysis

Wheat (*Triticum aestivum* L.) seeds of the Hanxuan10 variety were harvested following Natural Aging Treatment (NAT) in 2017 (8-year NAT) and 2024 (1-year NAT). Total RNA was extracted from these samples using the FastPure^®^ Universal Plant Total RNA Isolation Kit (Vazyme, Nanjing, China) following the manufacturer’s instructions. Full-length cDNA was synthesized using the HiScript IV All-in-One Ultra RT SuperMix (Vazyme). Quantitative real-time PCR (qPCR) analyses were conducted with the 2× Taq Pro Universal SYBR qPCR Master Mix (Vazyme) on the QuantStudio 6 Flex Real-Time PCR Systems (Thermo Fisher Scientific, Waltham, MA, USA). Each RT-qPCR assay included three technical replicates and was performed using three biologically independent RNA preparations. Relative gene expression was normalized to the internal reference gene *TaActin* and calculated using the 2^−ΔΔCT^ method, where CT represents the threshold cycle. Primer sequences are provided in Supplementary Table S2.

## 3. Results

### 3.1. Identification and Characterization of TaCRY Members

In this study, a total of twelve *TaCRY* members in wheat, alongside four *OsCRY* members in rice, five *ZmCRY* members in maize, and four *SiCRY* members in foxtail millet, were identified and characterized through a comprehensive genome-wide analysis, respectively. These *CRY* members were systematically named according to their highest sequence homology to *Arabidopsis AtCRY1*, *AtCRY2*, and *AtCRY3* orthologs (Supplementary Table S1).

Detailed physicochemical analyses revealed significant diversity among TaCRY proteins (Table 1). The amino acid (aa) lengths varied substantially, ranging from 593 residues in TaCRY3-7A to 712 residues in both TaCRY1-2A and TaCRY1-2B homeologs. Correspondingly, their relative molecular weights spanned 65,522.85 Da (TaCRY3-7D) to 80,626.7 Da (TaCRY1-2A). Theoretical isoelectric points (pI) exhibited a broad distribution from 5.31 (TaCRY2-6A) to 9.62 (TaCRY3-7D), suggesting varied protein charge states under physiological conditions. Further bioinformatics evaluations provided insights into protein stability and hydrophobicity. The instability index ranged from 41.17 (TaCRY2-6B) to 54.06 (TaCRY1-2B), indicating moderate to high stability across family members. The aliphatic index varied between 74.61 (TaCRY1-6B) and 85.49 (TaCRY2-6B), reflecting differential thermostability potentials. The grand average of hydropathicity values, ranging from -0.491 (TaCRY1-6D) to -0.328 (TaCRY2-6A), suggested overall hydrophilic characteristics typical of regulatory proteins. Subcellular localization predictions revealed distinct compartmentalization patterns: TaCRY1 and TaCRY2 proteins were predominantly predicted to localize in nuclear regions, while TaCRY3 members showed chloroplast-specific targeting. This differential distribution highlights potential functional divergence among TaCRY subfamilies.

### 3.2. Phylogenetic Analyses and Classification of TaCRY Members

**Figure 1:**
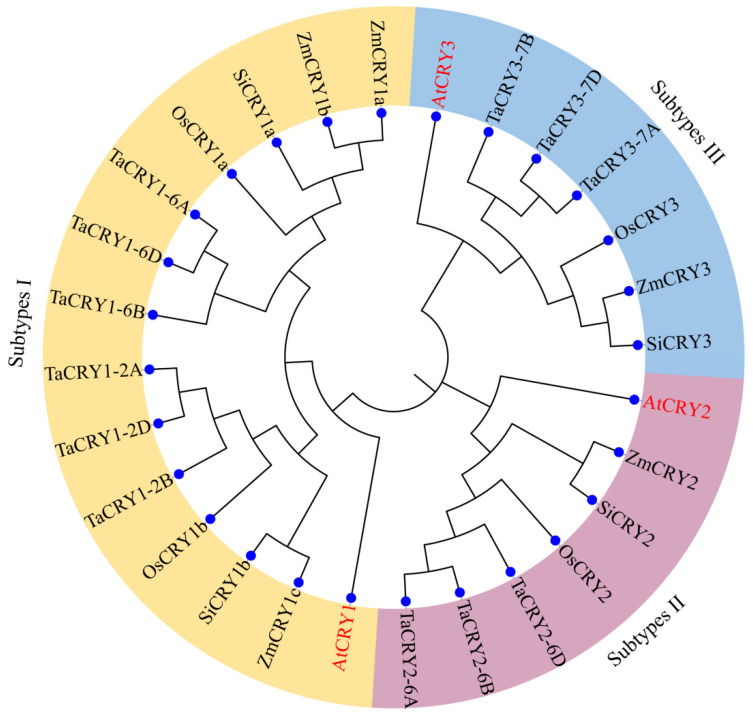
Phylogenetic tree of CRY proteins from *Arabidopsis thaliana*, wheat, rice, maize, and foxtail millet constructed using the neighbor-joining (NJ) method in MEGA 7.0.26. Each group is represented by different colors. At, *Arabidopsis thaliana*; Ta, *Triticum aestivum* L.; Os, *Oryza sativa* Japonica; Zm, *Zea mays* L.; Si, *Setaria italica* L.

To investigate the evolutionary relationships among the twelve *TaCRY* members, a phylogenetic tree was constructed using MEGA 7.0.26 software with the full-length amino acid sequences of three AtCRYs, four OsCRYs, five ZmCRYs, and four SiCRYs. Based on clustering with the well-characterized *AtCRY* subfamilies (*AtCRY1-3*), the *CRY1* subfamily exhibited the most complex composition in wheat, containing six *TaCRY1* paralogs compared to two *OsCRY1* orthologs in rice, three *ZmCRY1* in maize, and two *SiCRY1* in foxtail millet. The *CRY2* subfamily comprised three *TaCRY2* proteins, while the other grasses each possessed only one *CRY2* ortholog (*OsCRY2*, *ZmCRY2*, and *SiCRY2*). The *CRY3* subfamily was conserved as a single-copy ortholog in rice, maize, and foxtail millet (*OsCRY3*, *ZmCRY3*, and *SiCRY3*), but wheat contained three *TaCRY3* paralogs (Figure 1). Notably, the phylogenetic analysis reveals distinctive species-specific clustering patterns that delineate evolutionary relationships among these CRY proteins. TaCRYs and OsCRYs form a tightly clustered monocot-specific subclade, whereas AtCRY, ZmCRY, and SiCRY constitute a distinct dicot-associated lineage, reflecting the divergence of CRY proteins between monocotyledonous and dicotyledonous plants. Concurrently, the pronounced expansion of TaCRY members in wheat correlates with ancient whole-genome polyploidization events, suggesting that genetic redundancy may confer adaptive plasticity to diverse light environments. Furthermore, the marked phylogenetic distance separating the chloroplast-localized TaCRY3 subfamily from canonical nuclear-localized CRY proteins implies profound functional diversification within *CRY* gene family, potentially giving rise to novel regulatory pathways. Collectively, these evolutionary insights establish a critical phylogenetic framework for investigating conserved and divergent functional mechanisms governing CRY protein function across angiosperms.

### 3.3. Conserved Motifs, Domains, and Gene Structure Analysis of TaCRY Members

**Figure 2:**
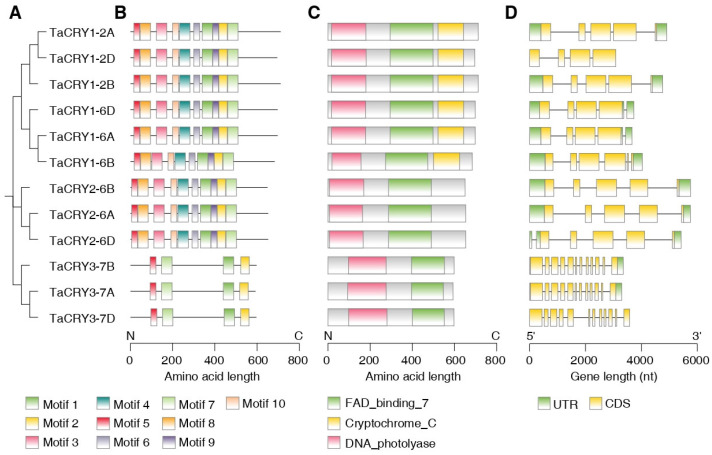
Phylogenetic relationship, conserved motif, conserved domain, and gene structure analysis of *TaCRY* members. (**A**) Phylogenetic tree. (**B**) Conserved motifs identified by MEME, with different colored boxes representing motifs containing specific amino acid sequences. (**C**) Domain architecture identified by NCBI-CDD, with different colored boxes representing conserved domains. (**D**) Exon/intron structures of *TaCRY* members.

To elucidate the structural diversity of *TaCRY* members, conserved motifs, domains, and gene structures were visualized in conjunction with a phylogenetic tree, enabling comparative analysis of evolutionary relationships and structural features (Figure 2A). Conserved motif analysis of *TaCRY* members using MEME identified 10 distinct motifs (motif 1–motif 10). Subtypes TaCRY1 and TaCRY2 retain all ten conserved motifs, suggesting a high degree of conservation in their core functional domains, whereas subtype TaCRY3 contains only four motifs (motif 1, motif 2, motif 5, and motif 7) (Figure 2B), indicating potential functional module simplification or divergence. Conserved domain analysis revealed distinct architectural features across CRY subfamilies. TaCRY1 proteins contain the canonical tripartite domain architecture characteristic of CRY proteins, comprising the DNA photolyase domain at the N-terminus, a central FAD-binding domain, and a unique signature Cryptochrome C domain. In contrast, TaCRY2 and TaCRY3 exhibit domain simplification, retaining only the DNA photolyase and FAD-binding domains while lacking the Cryptochrome C module (Figure 2C). This structural divergence suggests functional specialization through domain reduction. While the photolyase and FAD-binding domains likely mediate core photoreceptor activities conserved across all CRYs, the CRY1-specific C-terminal extension may confer subfamily-unique regulatory functions. The conservation of domain architecture across orthologous CRY1/CRY2/CRY3 groups suggests evolutionary pressure to maintain fundamental light-sensing machinery while allowing subfamily-specific structural diversification in terminal regions.

Gene structure analysis demonstrated significant heterogeneity among *TaCRY* members, with exon counts ranging from four to twelve. Specific exon configurations include: five exons in *TaCRY1-2A/2B*, four exons in *TaCRY1-2D/6A/6D*, six exons in *TaCRY1-6B*, five exons in *TaCRY2*, and a notable expansion to twelve exons in *TaCRY3* (Figure 2D). These structural variations suggest that *TaCRY* members have undergone functional differentiation and adaptive evolution during their evolutionary history, likely contributing to their distinct biological roles in light signal transduction pathways.

### 3.4. Tertiary Structure Models of TaCRY Members

**Figure 3:**
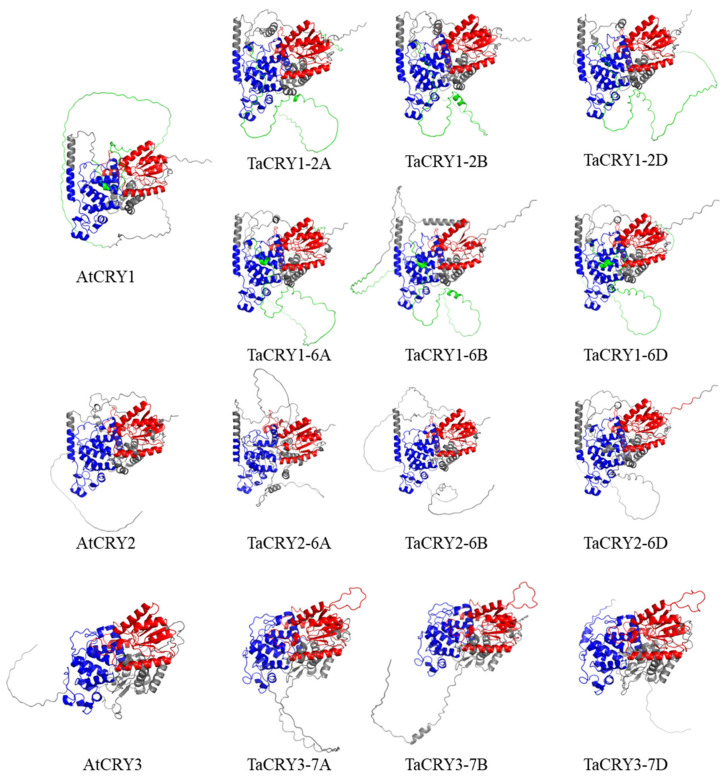
Predicted tertiary structures of CRY proteins from *Arabidopsis thaliana* and wheat using the AlphaFold 3. The structural model highlights three key domains: the DNA photolyase domain (red), the FAD-binding domain 7 (blue), and the Cryptochrome C-terminal extension (green). These domains are color-coded to illustrate their relative positions and functional regions within the protein architecture.

To elucidate the structural features of the CRY proteins, we employed the AI-driven AlphaFold 3 platform to predict the tertiary structures of *Arabidopsis* (AtCRYs) and wheat (TaCRYs) orthologs. Structural alignment revealed conserved folding patterns among proteins within the same CRY subfamily: AtCRY1 exhibited high structural homology with TaCRY1-2A, TaCRY1-2B, TaCRY1-2D, TaCRY1-6A, TaCRY1-6B, and TaCRY1-6D, while AtCRY2 showed structural conservation with TaCRY2-6A, TaCRY2-6B, and TaCRY2-6D. Notably, AtCRY3 demonstrated close structural similarity to TaCRY3-7A, TaCRY3-7B, and TaCRY3-7D but diverged significantly from the CRY1 and CRY2 subfamilies (Figure 3). This pattern indicates that cross-species orthologs within the same CRY subfamily maintain conserved tertiary structures, whereas paralogs from different subfamilies exhibit marked structural divergence.

### 3.5. Chromosomal Distribution and Collinearity Analysis of TaCRY Members

**Figure 4:**
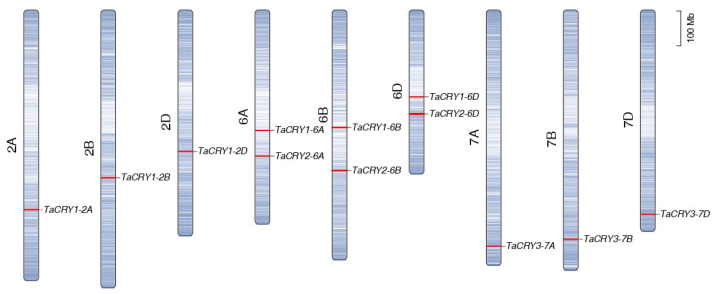
Chromosome distribution of *TaCRY* members in wheat.

**Figure 5:**
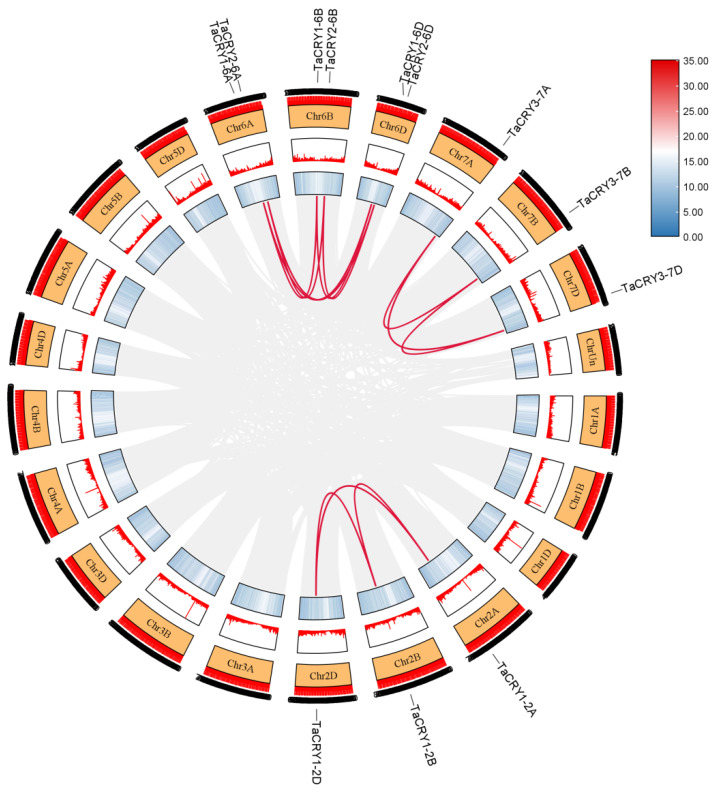
Collinearity analysis of *TaCRY* members. The red lines represent gene pairs with collinearity relationships, the numbers in the boxes represent chromosomes, and the red and light blue boxes represent gene density.

To elucidate the chromosomal distribution of *TaCRY* members, we conducted a comprehensive chromosomal localization analysis in wheat. This mapping revealed an uneven distribution of the 12 *TaCRY* members across chromosomes 2 (2A/2B/2D), 6 (6A/6B/6D), and 7 (7A/7B/7D) (Figure 4). Chromosomes 2A, 2B, 2D, 7A, 7B, and 7D each harbor one *TaCRY* gene. Notably, chromosomes 6A, 6B, and 6D each harbor two *TaCRY* genes, accounting for half of all characterized *TaCRY* members. This concentration suggests potential locus-specific regulatory or functional significance within this genomic region. The collinearity relationship analysis indicates that the expansion of the *TaCRY* gene family in wheat was primarily driven by whole-genome duplication events (Figure 5). For instance, the paralogous groups *TaCRY1-2A*, *TaCRY1-2B*, and *TaCRY1-2D*, as well as *TaCRY3-7A*, *TaCRY3-7B*, and *TaCRY3-7D*, demonstrate this duplication pattern. Additionally, the *TaCRY1-6A*, *TaCRY1-6B,* and *TaCRY1-6D* and *TaCRY2-6A*, *TaCRY2-6B,* and *TaCRY2-6D* subfamilies exhibit close evolutionary associations with the triplicated subgenomes of hexaploid wheat (Figure 5). These observations suggest that the ancestral *TaCRY* gene repertoire in progenitor wheat species (e.g., *Triticum urartu*) likely comprised only four foundational members, a configuration mirroring the *TaCRY* gene counts observed in other monocotyledonous species such as rice and foxtail millet. Collectively, this syntenic analysis provides preliminary insights into the evolutionary origins and conserved expansion mechanisms of the *TaCRY* gene family across grass species.

### 3.6. Genomic Landscape of Cis-Acting Elements in TaCRY Members

**Figure 6:**
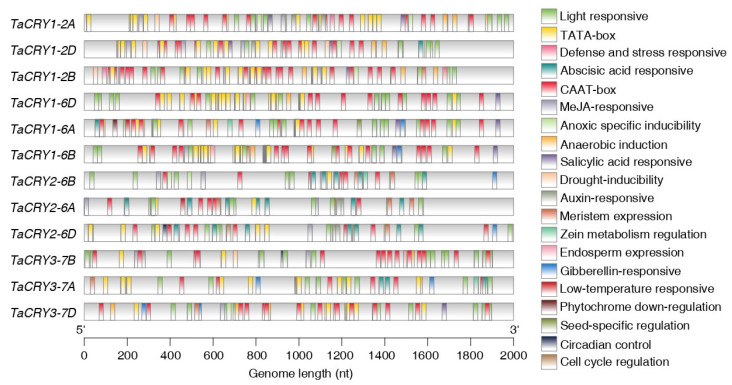
*Cis*-acting elements analysis of *TaCRY* members. Diverse colored boxes representing distinct *cis*-acting elements. Names of *cis*-acting elements are shown on the right.

The regulation of gene expression is primarily mediated by *cis*-acting elements within promoter regions located upstream of the transcription initiation site. To elucidate the expression patterns and regulatory mechanisms of *TaCRY* genes, we conducted a comprehensive analysis of *cis*-acting elements in the promoter regions of all 12 *TaCRY* members using the PlantCARE database. Our analysis revealed the presence of diverse functional *cis*-acting elements implicated in plant growth and development, phytohormone signaling, and responses to biotic/abiotic stresses (Figure 6). Beyond the core promoter elements (TATA-box and CAAT-box), we identified multiple regulatory elements associated with developmental processes. These include a light-responsive element, seed-specific regulation element, circadian control element, cell cycle regulation element, phytochrome down-regulation expression, meristem expression element, zein metabolism regulation element, and endosperm expression element. These findings suggest that TaCRY proteins participate in a broad spectrum of plant growth and development process. Furthermore, the promoter regions of *TaCRY* genes contain various phytohormone-responsive motifs, including a auxin-responsive element, gibberellin-responsive element, abscisic acid-responsive element, MeJA-responsive element, and salicylic acid-responsive element. The presence of these hormone-related *cis*-elements implies that *TaCRY* expression may be modulated through complex interactions with phytohormone signaling networks, thereby influencing developmental processes. Our analysis also predicted stress-related *cis*-acting elements, including defense and stress responsive elements, anoxic specific inducibility elements, anaerobic induction elements, drought inducibility elements, and low-temperature-responsive elements. These findings suggest that *TaCRY* members may have evolved specialized functions in coordinating biotic and abiotic stress responses, in addition to their roles in developmental regulation. Collectively, these observations indicate that the regulatory landscape of *TaCRY* promoters reflects functional diversification, enabling these genes to contribute to multiple biological processes through the integration of developmental cues, hormonal signals, and environmental stimuli.

### 3.7. Expression Analysis of TaCRY Members Related to Seed Vigor and Candidate Gene Identification

**Figure 7:**
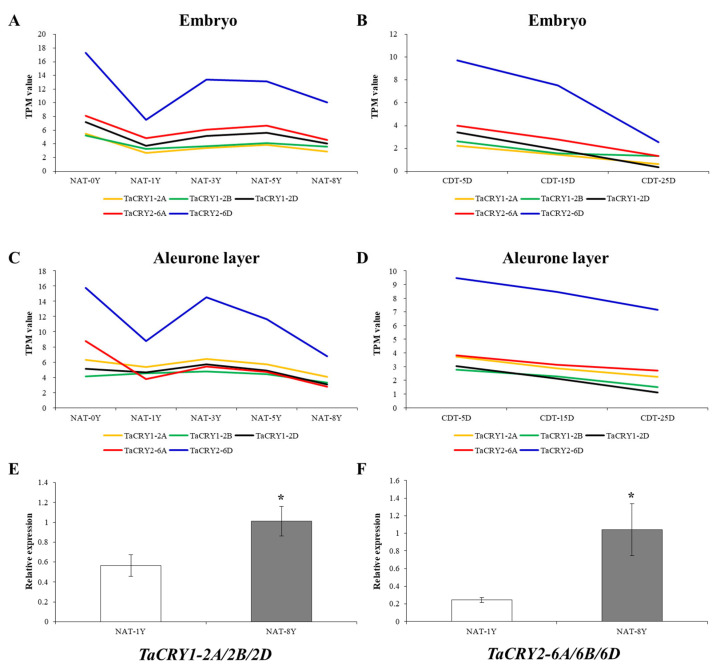
The expression of *TaCRYs* in embryo (**A**,**B**) and aleurone layer (**C**,**D**). Transcripts per million (TPM) value was used to normalize RNA-seq data. The expression of *TaCRY1-2A/2B/2D* (**E**) and *TaCRY2-6A/6B/6D* (**F**) in seeds; * represents *p*-value ≤ 0.05. The Y on the horizontal axis in Figures (**A**,**C**,**E**,**F**) represents year. The horizontal axis D in Figures (**B**,**D**) represents day.

To investigate the regulatory roles governing wheat seed vigor maintenance, we conducted comparative transcriptomic profiling of *TaCRY* gene family members across embryo and aleurone layer tissues from Chinese Spring seeds subjected to controlled deterioration treatment (CDT) and natural aging treatment (NAT). The Venn diagram showed that five *TaCRY* members display significant expression covariance with seed vigor (Supplementary Figure S1). Temporal expression dynamics revealed distinct regulatory patterns. While NAT-treated seeds exhibited gradual attenuation of *TaCRY* transcripts during aging (Figure 7A,C), CDT induced a more pronounced transcriptional repression. Notably, after 25 days of CDT exposure, *TaCRY* member expression levels were robustly diminished across both tissue types (Figure 7B,D). RT-qPCR analysis showed that the expression of *TaCRY1-2A/2B/2D* and *TaCRY2-6A/6B/6D* was significantly elevated under NAT-8Y compared to NAT-1Y (Figure 7E,F). These differential regulatory responses collectively imply that *TaCRY* genes may contribute to adaptive response processes influencing wheat seed vigor maintenance.

## 4. Discussion

Wheat, as one of the most critical global food crops, directly impacts grain yield and quality through seed vigor and quality [47]. During storage, wheat seeds undergo a series of physiological and biochemical changes due to environmental factors such as light, temperature, humidity, and oxygen concentration, leading to inevitable declines in seed vigor and quality. Reduced seed vigor not only affects germination and emergence rates but also results in weak seedling growth and poor stress resistance, ultimately compromising wheat yield and quality [48]. Although CRY-mediated regulation of seed dormancy and germination has been reported [31,32,49,50,51], systematic studies on the role of *TaCRY* members in regulating wheat seed vigor remain unclear. Therefore, a comprehensive investigation of the structural, functional, and expression patterns of the *TaCRY* gene family is essential for elucidating the regulatory mechanisms underlying wheat seed vigor.

### 4.1. Evolutionary Expansion and Functional Diversification of the TaCRY Members

The comprehensive characterization of the TaCRY gene family in wheat, alongside comparative analyses of *OsCRY* in rice, *ZmCRY* in maize, and *SiCRY* in foxtail millet, reveals evolutionary patterns and functional diversification. A total of 12 *TaCRY* members were identified. These genes were named, and their genomic positions, coding sequence lengths, and amino acid sequence lengths were recorded to establish foundational data for subsequent research. The expansion of *TaCRY* members correlates with ancient polyploidization events in wheat, suggesting that gene duplication has provided adaptive plasticity for light-responsive growth and development. Notably, the *TaCRY* gene family is partitioned into three distinct subfamilies (*TaCRY1*, *TaCRY2*, and *TaCRY3*) with clear evolutionary relationships to *CRY* genes in other species, each exhibiting conserved domain architectures and species-specific clustering patterns, suggesting both conservation and species-specific diversification during evolution. Furthermore, TaCRY1 and TaCRY2 form nuclear-localized orthologous groups with counterparts in other grasses, while TaCRY3 proteins represent a chloroplast-targeted lineage. Nuclear-localized TaCRY1 and TaCRY2 proteins are positioned to function as canonical blue-light receptors, modulating gene expression by interacting with COP1 and PIFs [1]. These genes are central hubs regulating light-responsive development, circadian rhythms, hormone biosynthesis, and stress responses [2]. Consequently, TaCRY1/2 subfamilies likely influence seed vigor by fine-tuning the expression of downstream genes involved in dormancy release, germination initiation, antioxidant defense, and hormone signaling during seed aging. In contrast, the chloroplast-targeted TaCRY3 subfamily represents a fascinating evolutionary adaptation, potentially unique to grasses. Its localization implies a primary role in perceiving light and redox status within the chloroplast [52,53]. Chloroplasts are major sites of reactive oxygen species (ROS) production during stress, including seed aging [54]. Therefore, TaCRY3 proteins may act as sentinels, directly sensing chloroplast stress (e.g., high light, oxidative burst) and potentially initiating retrograde signaling to the nucleus or modulating chloroplast-localized processes to maintain photosynthetic efficiency and mitigate oxidative damage during seed storage and early germination [55,56]. This functional specialization of TaCRY3 could be crucial for preserving seed energy reserves and combating ROS accumulation, key factors determining seed longevity and vigor [54].

The phylogenetic divergence between monocot (*TaCRYs* and *OsCRYs*) and dicot (*AtCRYs*) lineages reflects fundamental differences in light-signaling architectures. Monocot *CRYs* exhibit expanded paralog groups (e.g., six *TaCRY1* members vs. one *AtCRY1*), likely due to whole-genome duplication events. The monophyletic clustering of TaCRY3 with chloroplast-targeted proteins from other grasses suggests convergent evolution of organelle-specific CRY isoforms, potentially to optimize photosynthetic efficiency under high-light conditions. This structural functional paradigm shift from canonical nuclear CRYs to chloroplast-localized variants underscores the adaptive plasticity of the *CRY* gene family across plant lineages.

### 4.2. Structural Conservation and Subfamily-Specific Functional Modules

Gene structure analysis highlighted variations in exon–intron numbers and lengths among *TaCRY* genes, with some members exhibiting complex structures and others showing simpler organizations. This structural diversity likely correlates with functional divergence within the *TaCRY* members, as differential exon–intron combinations may influence gene expression regulation and protein function. Protein structure analysis underscores the evolutionary pressure to maintain core photoreceptor machinery while allowing subfamily-specific innovations. All TaCRY proteins retain the canonical DNA photolyase and FAD-binding domains, which are critical for light absorption and flavin cofactor interactions. However, structural divergence is evident in the C-terminal regions: TaCRY1 proteins possess a unique Cryptochrome C-terminal extension absent in TaCRY2/TaCRY3, which aligns with functional specialization in developmental regulation versus photoreceptor activity. This observation is supported by motif analysis, where TaCRY1 retains all 10 conserved motifs, while TaCRY3 exhibits a simplified motif composition, suggesting modular evolution of functional domains. The tertiary structure predictions further validate these findings, demonstrating conserved folding patterns within subfamilies but marked divergence between CRY1/CRY2 and CRY3, likely reflecting adaptation to distinct subcellular compartments (nuclear vs. chloroplast).

### 4.3. Functional Implications of the TaCRY Members for Wheat Seed Vigor

The *cis*-acting element analysis reveals a sophisticated regulatory landscape governing *TaCRY* expression, such as light-responsive, hormone-responsive, stress-related, and developmental elements. The prevalence of light-responsive elements and circadian regulation motifs aligns with *CRYs*’ canonical role as blue-light receptors. Light serves as a key environmental cue during germination, influencing dormancy release and seedling establishment. As blue-light receptors, TaCRY proteins perceive light signals and modulate downstream gene expression and physiological processes, such as regulating antioxidant enzymes, hormone biosynthesis, and metabolic pathways. These regulatory networks may alter redox states and hormone balances, thereby affecting germination potential and seedling vigor. However, the identification of hormone-responsive elements (GA, ABA) and stress-related motifs (drought, low-temperature) suggests that *TaCRYs* integrate multiple environmental cues to modulate growth–defense tradeoffs. Notably, the *TaCRY3* subfamily, despite lacking the Cryptochrome C domain, retains phytohormone-responsive elements, implying alternative regulatory mechanisms for chloroplast-localized functions. This regulatory complexity may enable wheat to fine-tune CRY activity under fluctuating field conditions, balancing energy production (via chloroplast signaling) with stress acclimation. The presence of ABA and GA response elements in *TaCRY* promoters may particularly be relevant to seed vigor. ABA promotes dormancy maintenance and inhibits germination, while GA antagonizes ABA to promote dormancy release and germination [23]. Seed aging is often associated with dysregulation of the ABA/GA balance, leading to delayed or reduced germination [57]. Nuclear-localized TaCRY1/2 members are prime candidates for modulating this hormonal crosstalk. Light signals perceived by TaCRY1/2 could influence the expression or activity of key enzymes in ABA (e.g., NCED-biosynthesis, CYP707A-catabolism) and GA (e.g., GA20ox-biosynthesis, GA2ox-catabolism) metabolism or directly regulate signaling components like PYR/PYL ABA receptors or DELLA repressors [24]. We hypothesize that specific TaCRY1/2 paralogs may function similarly in wheat seeds, and their altered expression during aging (as observed for some members) might contribute to restoring or disrupting the hormonal equilibrium critical for germination capacity.

The expression profile linking *TaCRY* genes to seed vigor highlights their agronomic relevance. Five *TaCRY* members exhibited differential expression during seed aging, with co-localization analysis suggesting their involvement in maintaining germination potential under stress. This aligns with the identified *cis*-elements for seed-specific regulation and abiotic stress responses, proposing that *CRYs* modulate seed longevity through antioxidant pathways or hormonal crosstalk. For instance, during seed aging, ABA/GA balance disruption may deepen dormancy or reduce germination capacity [29]. TaCRY proteins might mitigate this by modulating hormone biosynthesis genes. Similarly, stress-responsive elements imply roles in oxidative stress tolerance, where *TaCRY* genes may activate antioxidant defenses to scavenge reactive oxygen species (ROS) and maintain seed integrity. Developmental elements further indicate potential involvement in seed maturation processes. These findings position *TaCRYs* as candidate targets for improving seed quality in wheat breeding programs, particularly under climate change scenarios where accelerated seed aging threatens food security.

This study provides comprehensive insights into the *TaCRY* gene family and suggests its potential implications in processes related to wheat seed vigor. Defining the precise regulatory mechanisms of *TaCRY* members requires further functional validation. Subsequent utilization of advanced breeding technologies, including gene editing or transgenic approaches, targeting validated *TaCRY* genes holds promise for developing wheat varieties exhibiting improved storage stability, germination rates, and stress resilience, ultimately contributing to sustainable agricultural productivity.

## 5. Conclusions

This study conducted a comprehensive genome-wide characterization of the *TaCRY* gene family in wheat, and systematically analyzed their phylogenetic relationships, gene structures, conserved domains, collinearity, *cis*-acting elements, and transcriptional responses to wheat seed vigor during seed aging. The results revealed that the *TaCRY* members had diverse structural characteristics in the wheat genome. The expression of *TaCRY* members is subject to complex regulatory networks involving both endogenous and exogenous signals. It also has a significant transcriptional response to wheat seed vigor during the seed aging process. Collectively, these findings have established a functional genomics foundation for elucidating the molecular mechanisms underlying *TaCRY*-mediated regulation of wheat seed vigor, with significant implications for genetic improvement strategies in wheat breeding programs.

## Figures and Tables

**Table 1:** Physicochemical properties of *TaCRY* members.

Gene Name	AA (aa)	MW (Da)	pI	Instability Index	Aliphatic Index	GRAVY	Subcellular Localization
*TaCRY1-2A*	712	80,626.7	5.54	53.09	76.03	−0.428	nucleus
*TaCRY1-2B*	712	80,497.49	5.41	54.06	76.43	−0.41	nucleus
*TaCRY1-2D*	696	78,841.74	5.58	52.95	77.08	−0.412	nucleus
*TaCRY1-6A*	698	79,033.49	5.34	51.53	73.8	−0.475	nucleus
*TaCRY2-6A*	653	73,450.14	5.31	43.87	85.25	−0.328	nucleus
*TaCRY1-6B*	684	77,644.21	5.55	51.61	74.61	−0.465	nucleus
*TaCRY2-6B*	650	73,258.94	5.62	41.17	85.49	−0.363	nucleus
*TaCRY1-6D*	698	79,049.55	5.52	52.02	74.63	−0.491	nucleus
*TaCRY2-6D*	653	73,643.45	5.44	44.96	83.91	−0.351	nucleus
*TaCRY3-7A*	593	65,929.05	9.44	44.01	77.69	−0.389	chloroplast
*TaCRY3-7B*	598	66,333.77	9.56	43.98	79.65	−0.346	chloroplast
*TaCRY3-7D*	597	65,522.85	9.62	41.73	76.68	−0.376	chloroplast

Note: The last digit and letter of the gene names represent chromosome numbers. Abbreviations: AA (aa), number of amino acids; MW (Da), molecular weight (Daltons); pI, theoretical isoelectronic point; GRAVY, grand average of hydropathicity.

## Data Availability

Data are contained within the article.

## Supplementary Materials

The supporting information can be downloaded at: https://www.mdpi.com/article/10.3390/cimb47070522/s1.
