# Supplementary material for: Genome-Wide Identification and Characterization of TaCRY Gene Family and Its Expression in Seed Aging Process of Wheat
Source: Curr Issues Mol Biol. 2025 Jul 6;47(7):522. doi: 10.3390/cimb47070522 (PMC12293649; doi:10.3390/cimb47070522)
Supplement: Supplementary file 1 [file cimb-47-00522-s001.zip › cimb-3682028-supplementary.pdf]

Supplementary Table S1 CRY members rename

**Gene ID**	**Rename**
TraesCS2A02G340000.1	TaCRY1-2A
TraesCS2B02G337900.1	TaCRY1-2B
TraesCS2D02G317500.1	TaCRY1-2D
TraesCS6A02G203300.1	TaCRY1-6A
TraesCS6A02G223700.1	TaCRY2-6A
TraesCS6B02G224100.2	TaCRY1-6B
TraesCS6B02G257600.2	TaCRY2-6B
TraesCS6D02G187200.1	TaCRY1-6D
TraesCS6D02G211700.1	TaCRY2-6D
TraesCS7A02G489800.1	TaCRY3-7A
TraesCS7B02G392900.2	TaCRY3-7B
TraesCS7D02G476200.1	TaCRY3-7D
AT4G08920.1	AtCRY1
AT1G04400.1	AtCRY2
AT5G24850.1	AtCRY3
Os02t0573200-01	OsCRY1a
Os02t0625000-01	OsCRY2
Os04t0452100-02	OsCRY1b
Os06t0661800-01	OsCRY3
Zm00001eb081200_P004	ZmCRY1c
Zm00001eb182820_P002	ZmCRY1a
Zm00001eb244770_P002	ZmCRY1b
Zm00001eb382070_P001	ZmCRY2
Zm00001eb389640_P002	ZmCRY3
KQK97517	SiCRY1b
KQL10631	SiCRY2
KQL11079	SiCRY3
KQL30144	SiCRY1a

Supplementary Table S2 Primer sequences used in this study

Name	Primer sequences	
TaCRY1-2A/2B/2D-F	GGCAGTACATCTCTGGCTC	RT-qPCR
TaCRY1-2A/2B/2D-R	GCTGTATCTAGCTCAACTATG	
TaCRY2-6A/6B/6D-F	GGCAGGAATCTTGAGTTAC	
TaCRY2-6A/6B/6D-R	GCACTTCGACTGAAGATG	
TaActin-F	CCTTCAGTTGCCCAGCAATGT	
TaActin-R	CATTAGATTATCCGTGAGGTC	

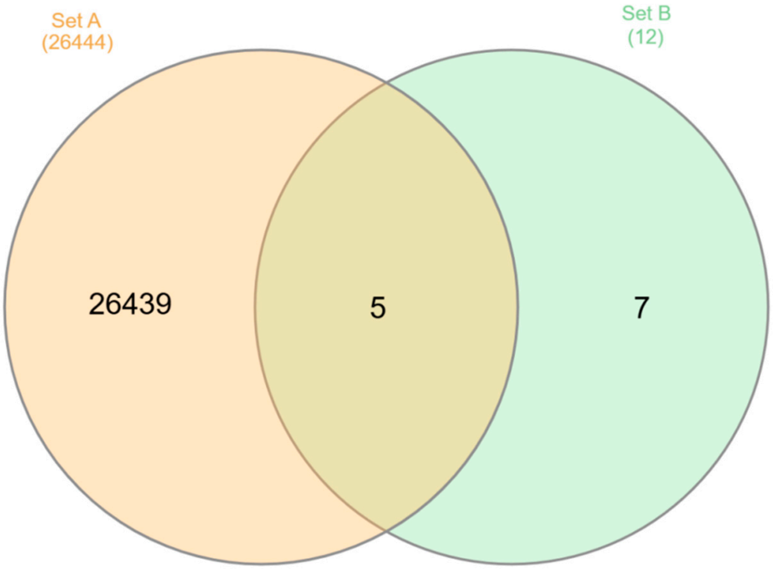

Supplementary Figure S1 Venn diagram showing five TaCRY members associated with wheat seed vigor. Set A represents differentially expressed genes (DEGs) associated with wheat seed vigor. Set B represents TaCRY members.
